# Dynamics of loops surrounding the active site architecture in GH5_2 subfamily *Tf*Cel5A for cellulose degradation

**DOI:** 10.1186/s13068-023-02411-2

**Published:** 2023-10-18

**Authors:** Xiuyun Wu, Sha Zhao, Zhennan Tian, Chao Han, Xukai Jiang, Lushan Wang

**Affiliations:** 1 State Key Laboratory of Microbial Technology, Institute of Microbial Technology, Shandong University, Qingdao, 266237 China; 2 National Glycoengineering Research Center, Shandong University, Qingdao, 266237 China; 3 Shandong Key Laboratory of Agricultural Microbiology, Shandong Agricultural University, Tai’an, 271018 China

**Keywords:** Cellulase, GH5_2 subfamily, Active site architecture, Loop dynamics, Enzyme catalysis

## Abstract

**Background:**

Lignocellulose is the most abundant natural biomass resource for the production of biofuels and other chemicals. The efficient degradation of cellulose by cellulases is a critical step for the lignocellulose bioconversion. Understanding the structure-catalysis relationship is vital for rational design of more stable and highly active enzymes. Glycoside hydrolase (GH) family 5 is the largest and most functionally diverse group of cellulases, with a conserved TIM barrel structure. The important roles of the various loop regions of GH5 enzymes in catalysis, however, remain poorly understood.

**Results:**

In the present study, we investigated the relationship between the loops surrounding active site architecture and its catalytic efficiency, taking *Tf*Cel5A, an enzyme from GH5_2 subfamily of *Thermobifida fusca*, as an example. Large-scale computational simulations and site-directed mutagenesis experiments revealed that three loops (loop 8, 3, and 7) around active cleft played diverse roles in substrate binding, intermediate formation, and product release, respectively. The highly flexible and charged residue triad of loop 8 was responsible for capturing the ligand into the active cleft. Severe fluctuation of loop 3 led to the distortion of sugar conformation at the − 1 subsite. The wobble of loop 7 might facilitate product release, and the enzyme activity of the mutant Y361W in loop 7 was increased by approximately 40%.

**Conclusion:**

This study unraveled the vital roles of loops in active site architecture and provided new insights into the catalytic mechanism of the GH5_2 cellulases.

**Supplementary Information:**

The online version contains supplementary material available at 10.1186/s13068-023-02411-2.

## Introduction

Cellulose, an insoluble linear polysaccharide composed of d-glucose units linked by β-1,4-glycosidic bonds, is the most abundant natural biopolymer on earth and ranges in length from 2000 to 25,000 glucose residues [1]. A crucial factor that prevents the conversion of cellulose to other molecules is the recalcitrant nature of its crystalline region [2]. The complete breakdown of cellulose requires the synergistic hydrolytic action of three main enzymes: β-1,4-endoglucanase (EC 3.2.1.4), cellobiohydrolase (exoglucanase; EC 3.2.1.91 and EC 3.2.1.176), and β-glucosidase (EC 3.2.1.21) [3]. The crystalline surface of cellulose can also be disrupted by the oxidative action of lytic polysaccharide monooxygenases [4]. Among these enzymes, β-1,4-endoglucanase plays a key role in cellulose degradation. According to the Carbohydrate-Active enZYmes database (CAZy; http://www.cazy.org/), β-1,4-endoglucanases are classified into glycoside hydrolase (GH) families 5, 6, 7, 8, 9, 10, 12, 26, 44, 45, 48, 51, 124, and 148, which randomly cleave internal glycosidic bonds in amorphous cellulose [5]. Several cellulases have been used as fundamental ingredients in industrial enzyme cocktails to degrade cellulose; however, the intrinsic instability and low catalytic activity of cellulases are the major hurdles in their widespread application in biorefinery industries [6, 7]. Therefore, it is essential to explore the catalytic mechanism of cellulases for efficient cellulose conversion [8, 9].

The GH5 family is one of the largest GH families and the first cellulase family to be described, and it is historically known as “cellulase family A” [10]. The GH5 family has a long evolutionary history, and its members are widely distributed in the kingdoms of bacteria, archaea, and eukaryotes. In recent years, with the accumulation of sequence data, a more detailed subfamily classification has emerged in the GH5 family [11]. In particular, a new GH5 subfamily classification system has been proposed based on the criterion of sequence identity > 75%, and in this new system, 51 subfamilies cover more than 80% of the sequences. Currently, there are 56 subfamilies in the GH5 family according to their phylogenetic relationships. β-1,4-endoglucanases are the key members of the GH5 family and are distributed in nine major subfamilies (GH5_1, 5_2, 5_4, 5_5, 5_22, 5_25, 5_26, 5_37, and 5_39 subfamilies). Some subfamilies of enzymes were multifunctional, indicating that mutations of only a few amino acids could lead to functional divergence [11]; this feature was also confirmed by the modification of the GH5_4 subfamily [12]. Therefore, the structural basis of substrate recognition has become a promising area of research as scientists are seeking to further understand the functional diversity of the GH5 family [13, 14].

GH5 enzymes possess a highly conserved overall structure, namely a canonical (β/α)_8_ TIM barrel fold with eight loops surrounding the catalytic cleft [15]. This structural fold provides abundant freedom in variation for the loops, which leads to the catalytic diversity of the proteins, including substrate specificity, hydrolytic activity, thermostability, and optimal pH [16]. Recently, several research studies have focused on investigating the connecting loop regions of enzymes to improve the performance of enzyme molecules. The structural arrangements of loops in the GH5_4 subfamily was found to contribute to the breadth of substrate selectivity and hydrolytic reactions [17]. Zheng et al. enhanced the catalytic activity of *Gt*Cel5 by mutating the asparagine on loop 6 to alanine or glycine, and the molecular dynamics (MD) simulation suggested that the increased flexibility of loop 6 improved the interactions between the enzyme and the substrate [5]. The loops have also been observed to affect the catalytic properties of enzymes in other GH families. For instance, Yang et al. revealed that the longer loop 3 of GH12 endoglucanases might strengthen the hydrogen network of the active site architecture and thus increase the catalytic efficiency of the enzymes [18]. Designing the flexible region of enzymes could counteract the stability-activity trade-off and enhance the thermostability and catalytic activity [19, 20].

Loop conformational dynamics, particularly “lid loop,” play an important role in the process of substrate selection and recognition as well as in mediating substrate entry into the active clefts of the enzyme [21, 22]. Previous studies have shown that the difference between the state of the enzyme and substrate before and after binding lies mainly in the conformational difference of the loop structure in the active center region [23]. To elucidate how loops in the active site architecture regulate the catalytic activity of enzymes, the structure and dynamics of the loops should be studied. Given the limitations of using experiments alone to probe this aspect, MD simulations provide a visual model to show possible connections between the structure and dynamics of loops by exploring various conformational ensembles. We previously performed MD simulations under continuous systems to investigate changes in the protein dynamics of *Tf*Cel5A during substrate binding; the findings indicated that conformational imbalance caused by substrate binding was likely to be an important trigger of attenuation observed in cellulose hydrolysis [24]. In another study, we identified a salt-sensitive loop in GH5_2 enzymes using MD simulations, which facilitated the rational design of novel halotolerant enzymes [25]. Moreover, the flexibility of the tunnel-forming loops could be significantly affected by pH, thereby resulting in changes in processivity and substrate complexation [26].

In the present study, we integrated structural bioinformatics, MD simulations, and site-directed mutagenesis to investigate the role of loops in the active site architecture of the GH5_2 cellulase *Tf*Cel5A, an enzyme from *Thermobifida fusca*. The experiments conducted at atomic and molecular scales revealed that loops 3, 4, 7, and 8 played different roles in enzymatic catalysis, such as initial substrate binding, intermediate formation, and product release. Interestingly, the functions of these loops were highly dependent on their unique structural dynamics following interaction with the cellulose substrate. These findings provided mechanistic insights into the catalytic activity of GH5_2 cellulases and a new mechanism-based strategy to rationally design cellulases for biomass conversion.

## Results

### Loop features of the active site architecture of GH5_2 cellulases

**Fig. 1:**
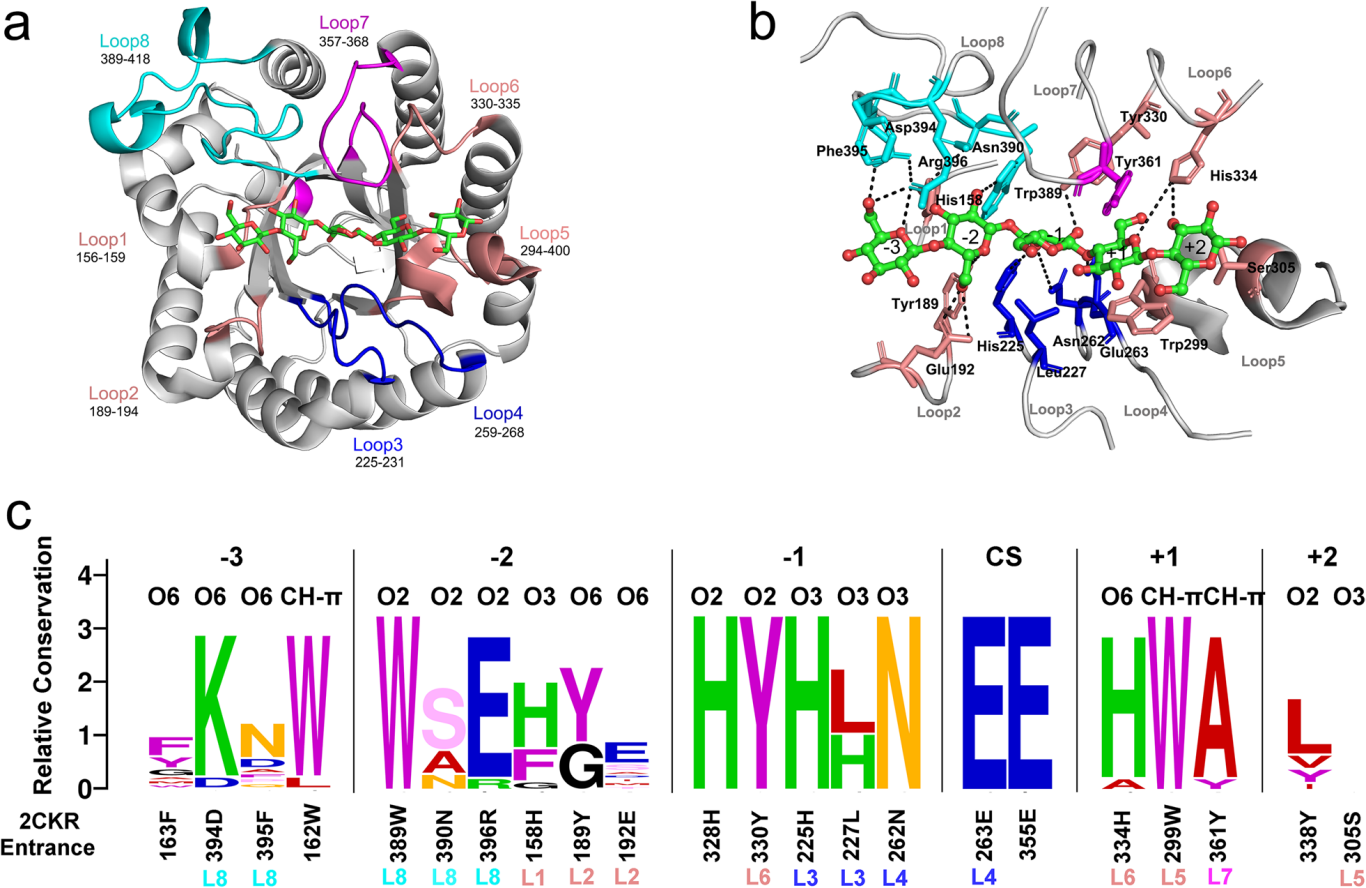
Loop analysis of the active site architecture of GH5_2 cellulases. **a** *Tf*Cel5A-cellopentose complex. The loops of the active site architecture are shown in colors. Loops 1, 2, 5, and 6 are shown in salmon; loops 3 and 4 are shown in blue; and loops 7 and 8 are shown in magenta and cyan, respectively. Cellopentose in the active cleft is shown in green. **b** Hydrogen bond network between the residues in loops 1–8 and the ligand. The subsites of the active cleft are labeled from − 3 to + 2. **c** Sequence profile of the active site architecture of GH5_2 enzymes. Based on the cut-off of 5 Å, the amino acid residues surrounding cellopentose in the *Tf*Cel5A-cellopentose complex were selected to generate the reference using PyMOL software. The size of the letter indicates the degree of conservation of the residues. The sugar atoms interacting with the enzyme are labeled above the residue

Based on the CAZy database, six subfamilies of the GH5 family were analyzed for evolution. Among these subfamilies, the GH5_2 subfamily had the largest number of characterized enzymes and crystalline structures (Additional file 1: Fig. S1). To show the loop features of the active site architecture of GH5_2 cellulases, a representative cellulase *Tf*Cel5A (PDB: 2CKR) from *T. fusca* was used as the reference [24, 25]. The crystalline structure of *Tf*Cel5A was solved with a cellopentose chain in its active cleft. As shown in Fig. 1a, the active site architecture comprised eight loops, named loop 1 to loop 8 according to the number of residues. The entire active site was divided into five subsites, spanning from − 3 subsite (non-reducing end) to + 2 subsite (reducing end) according to the cleaving site of the cellulose chain. The interaction network between 17 amino acid residues in the loops and cellopentose was analyzed (Fig. 1b). The catalytic residues were 263E and 355E, with 263E located on loop 4. Bortoli-German et al. clarified that Glu133 and Glu220 in cel5A from *Bacillus agaradhaerens* served as proton donors and nucleophiles, respectively, to form glycosylase intermediates [27]. The five amino acids (394D, 395F, 389W, 390N, and 396R) of loop 8 formed hydrogen bond interactions with the sugar rings at the − 3 and − 2 positions. In addition, hydrogen bond interactions with amino acids at the − 2 subsite also included 158H at loop 1 and 189Y and 192E at loop 2. The amino acids 225H at loop 3, 227L at loop 6, 330Y at loop 4, and 262N at loop 4 formed interactions with the − 1 sugar ring. At the + 1 and + 2 subsites, the amino acids 299W and 305S at loop 5, 334H at loop 6, and 361Y at loop 7 formed interactions with the ligands. Moreover, the hydrogen-bonding networks at − 3 to − 1 subsites were roughly the same as that of cel5A from *Bacillus agaradhaerens* analyzed by atomic resolution [28]. The difference is that the polar amino acids that interact with the − 3 and − 2 sugar rings are 267K, 269E in cel5A, while 394D and 396R in *Tf*Cel5A.

To validate the generality of the significance, we used a structural bioinformatics method to analyze the evolution of the active site residues in the GH5_2 subfamily (Fig. 1c). The amino acid residues at the − 1 and − 2 subsites were more conserved in the family than those at the − 3, + 1, and + 2 subsites. Among these residues, 225H at loop 3 (L3), 262N and 263E at loop 4 (L4), 299W at loop 5 (L5), 330Y at loop 6 (L6), and 389W at loop 8 (L8) were absolutely conserved in the GH5_2 subfamily. Moreover, 394D and 396R at loop 8, 334H at loop 6, and 361Y at loop 7 (L7) were relatively conserved. In cel5A, conserved 98H (225H in *Tf*Cel5A), 192H (328H in *Tf*Cel5A) and 198H (334H in *Tf*Cel5A) were proved to be essential for the catalytic activity of the enzyme, but do not affect its in vivo stability, while non-conserved Arg residues played an important role in protein stabilization [27, 29]. In addition, our previous results suggested that alanine substitution of conserved residues of xylanases from GH10 and GH11 family lead to a significant decrease in enzymatic activity [30, 31].

### Analysis of flexibility of loops in *Tf*Cel5A

**Fig. 2:**
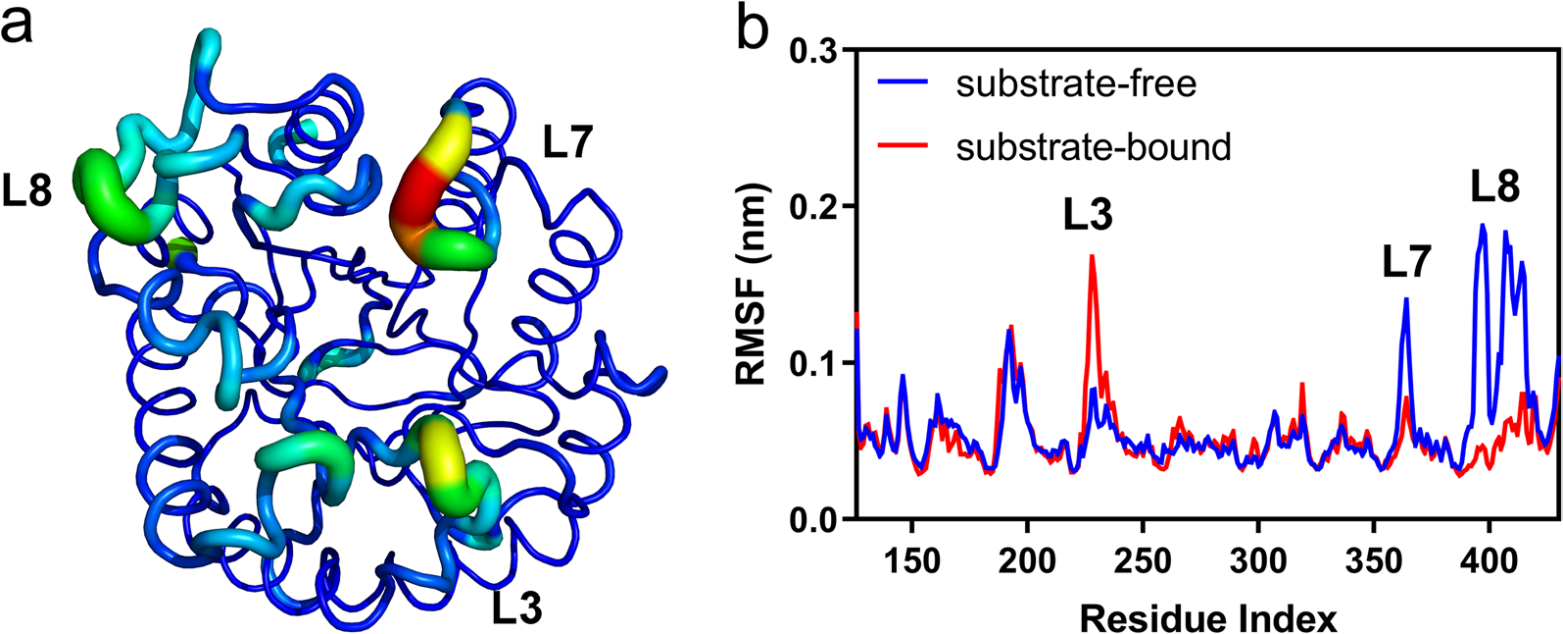
Structural flexibility of *Tf*Cel5A. **a** B-factor analysis of the entire structure of *Tf*Cel5A. Visualizing relative disorder or uncertainty in atomic positions is done by coloring by temperature value. Atoms with low temperature values are colored blue, while atoms with high temperature values are colored red. **b** Comparison of the RMSF values between the substrate-free (blue line) and substrate-bound states (red line). Locations of loops 3, 7, and 8 are labeled

To further investigate the structural flexibility of *Tf*Cel5A, particularly the dynamics of key loops of the active site architecture, we performed b-factor analysis and RMSF calculation. The b-factor values reflect the “diffusion” of the electron density of the atoms in the crystal, which actually reflects the conformational state of the protein molecule in the crystal [32]. The b-factors of *Tf*Cel5A (PDB: 2CKR) overall structure were shown in Fig. 2a, and represented by the thickness and color of the displayed tubes. The b-factor values of loops 3, 7, and 8 were high, indicating that these three loops have high flexibility. We also calculated the root mean square fluctuation (RMSF) changes for the two simulation systems: substrate-free state and substrate-bound state. Although the RMSF values of most protein regions remained almost unchanged, the RMSF values of several regions increased and decreased significantly. In particular, three regions (loops 3, 7, and 8) always showed the largest RMSF change between the substrate-free and substrate-bound states (Fig. 2b). Moreover, the corresponding loops in several other GH5_2 enzymes exhibited similar structural dynamics (Additional file 1: Fig. S2). This indicated that the unique dynamics of these loops might be a key factor in the catalysis of GH5 enzymes. Importantly, our large-scale MD simulations of 21 enzymes from 6 different GH5 subfamilies revealed that loop 7 and 8 always showed high flexibility (Additional file 1: Fig. S3).

### Loop 8 mediated the initial substrate binding

**Fig. 3:**
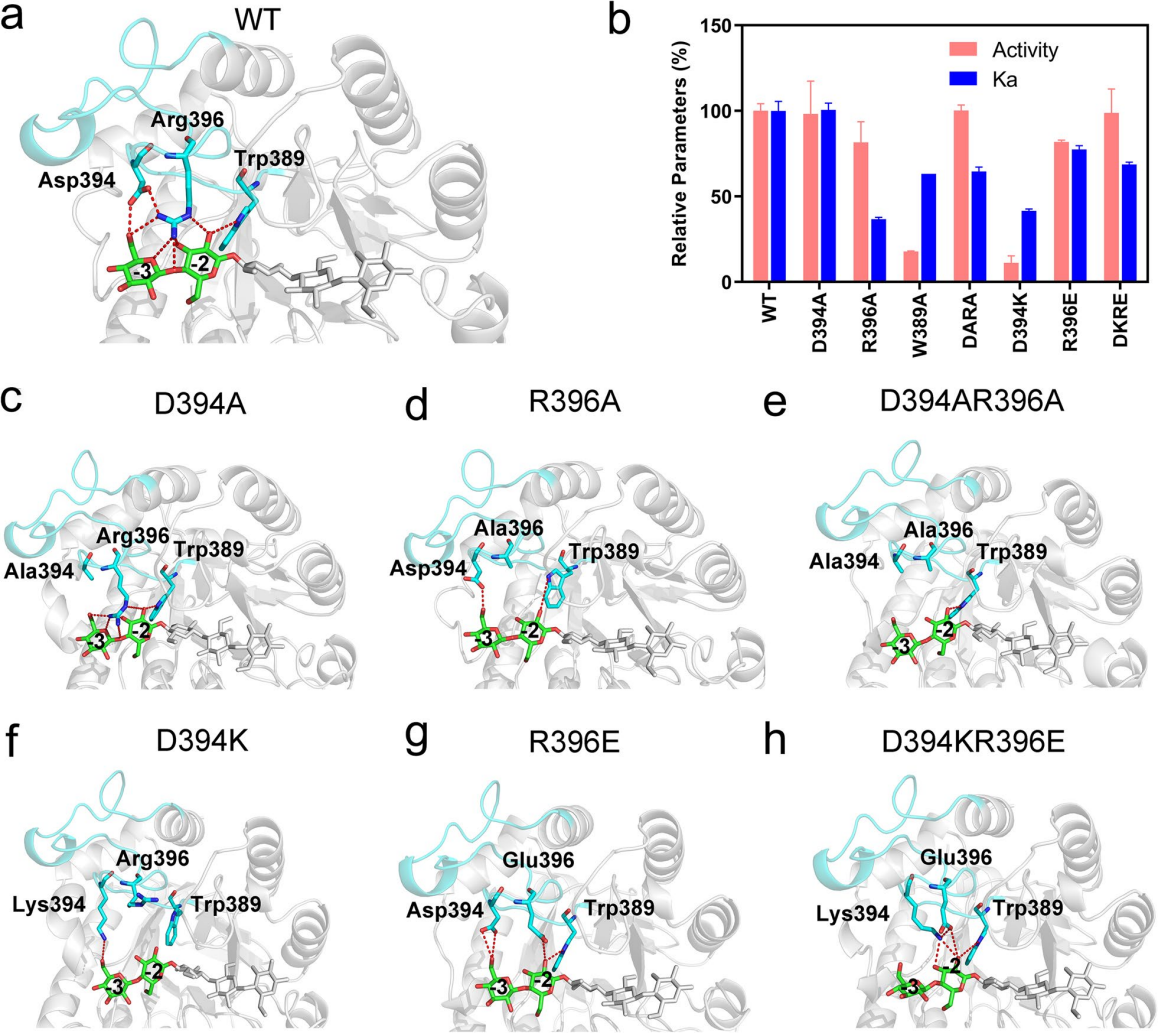
Interaction between residues in loop 8 and the substrate. **a** The hydrogen bonding network between wild-type *Tf*Cel5A and the substrate at the − 3 and − 2 subsites. **b** Measurements of the catalytic activity relative to WT *Tf*Cel5A and substrate binding affinity of the loop 8 mutants. DARA and KDRE represent double mutants D394A_R396A and D394K_R396E, respectively. **c**–**h** The hydrogen bonding interactions of loop 8 and the substrate revealed by the MD simulations of the mutants

Interestingly, according to local structural analysis, loop 8 contains an acidic amino acid (394D in *Tf*Cel5A) and a basic amino acid (396R in *Tf*Cel5A) that appeared adjacent to the fully conserved 389W (Fig. 3a and Additional file 1: Fig. S4). The residue triad in loop 8 was located at the non-reducing end of the active site and formed extensive interactions with the sugars at the − 3 and − 2 subsites. 394D formed a hydrogen bond with 396R and a hydrogen bond with the O6ʹ-hydroxyl group of glucose at the − 3 subsite; 396R formed four hydrogen bonds with glucose at the − 3 and − 2 subsites; and 389W formed a hydrogen bond with glucose at the − 2 subsite. Considering the functional importance of the loops in enzymes with a TIM barrel fold [5, 33, 34], we hypothesized that the residue triad in loop 8 was likely to be a unique motif essentially required for the catalysis of *Tf*Cel5A. Therefore, we constructed a series of mutants with mutations at positions 394, 396, and 389 and measured the substrate binding affinity and catalytic activity of the mutants (Fig. 3b). Alanine substitution of 389W sharply decreased the catalytic activity of *Tf*Cel5A by 82.2%, which reflected the critical role of 389W in loop 8 of *Tf*Cel5A. Single mutants D394A and R394A and the double mutant D394A_R394A showed catalytic activities similar to that of the wild type (WT). However, the catalytic activity and substrate binding affinity of D394K significantly decreased by 82.3% and 59.4%, respectively. In contrast, the catalytic activity and substrate binding affinity of R396E decreased by approximately 20%. Interestingly, when we swapped the electrochemical properties at positions 394 and 396, the double mutant D394K_R396E showed similar catalytic activity to the WT. Collectively, these results indicated that the unique residue motif in loop 8 played a vital role in substrate binding and catalytic activity of *Tf*Cel5A. The enzymatic activities and binding force changes of the other alanine mutations that interact with the sugars at positions − 3 and − 2 are shown in Additional file 1: Fig. S5. Following alanine mutation, the enzyme activities remained above 60% of the activity of the wild-type enzyme, while the enzyme activities of 395F and 192E remained unchanged.

To further investigate the functional mechanism of the residue triad in loop 8, we performed a series of MD simulations for each mutant. The simulations with D394A showed that 386R and 389W formed extensive hydrogen bonding interactions with the substrate (Fig. 3c) and this finding explained the experimentally measured non-significantly different activity and binding affinity between WT and D394A. In the simulations with R396A, 394D and 389W formed two hydrogen bonds with the sugars (Fig. 3d), resulting in a decrease in its substrate-binding affinity. In the simulations with D394A_R396A, only 389W formed a hydrogen bond with the sugar at the − 2 subsite (Fig. 3e), while the activity of D394A_R396A surprisingly remained similar to that of the WT. In the simulations with D394K, the electrostatic repulsion between 394 K and 396R changed the orientation of the sidechains of both 396R and 389W (Fig. 3f); this perturbed the hydrogen bonding network at the − 2 and − 3 subsites and significantly decreased the catalytic activity of D394K. In the simulations with R396E and D394K_R396E, loop 8 maintained an extensive hydrogen bonding network with the substrate (Fig. 3g and h), which might account for their similar activities to the WT. The unique structural dynamics enabled the residue triad (i.e., 389W, 394D, and 396R in *Tf*Cel5A) to search for the substrate in the solution and finally captured the cellulose chain into the active cleft of GH5_2 cellulases.

### Loops 3 and 4 promoted the formation of the intermediate

**Fig. 4:**
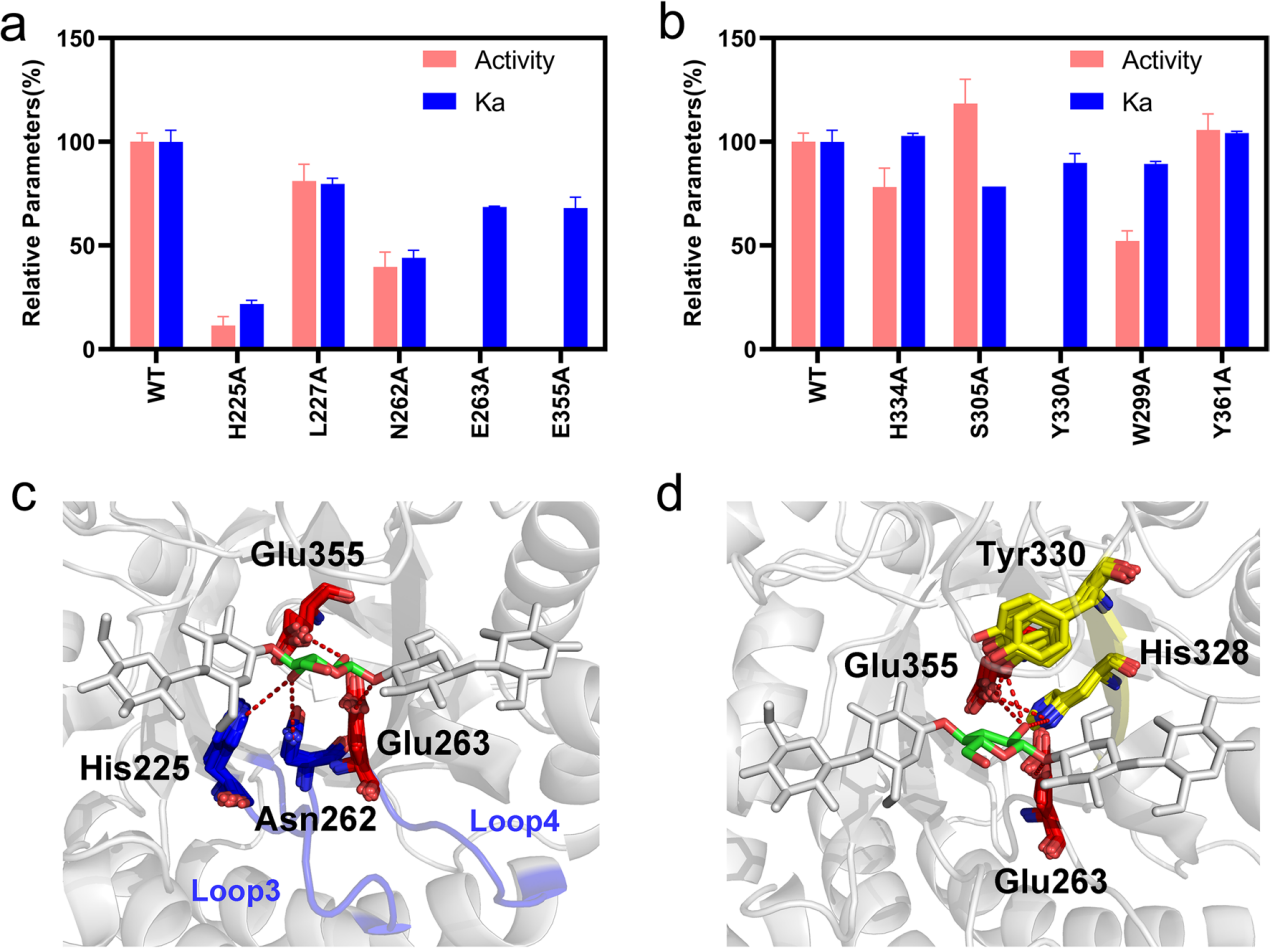
Functional analysis of key residues in loops 3, 4, and 6. **a** Measurements of the catalytic activity relative to WT *Tf*Cel5A and substrate binding affinity of loop 3 and 4 mutants. **b** Measurements of the catalytic activity relative to WT *Tf*Cel5A and substrate binding affinity of loop 5, 6, and 7 mutants. **c** Hydrogen bonding interaction between the active site residues and the O3ʹ-hydroxyl group of the sugar at the − 1 subsite. **d** Hydrogen bonding interaction between the active site residues and the O2ʹ-hydroxyl group of the sugar at the − 1 subsite

To discover the functional determinants of the − 1 and + 1 subsites in the active site architecture, we constructed mutants with alanine-scanning substitutions and measured the substrate binding affinity and catalytic activity of the mutants (Fig. 4a and b). Compared to the wild-type *Tf*Cel5A, the alanine substitutions at almost all positions decreased the substrate binding affinity and catalytic activity of the mutants, which confirmed the significance of the active site residues in catalysis. Notably, the alanine substitutions at the − 1 subsite dramatically decreased the catalytic activities by 88.5% (H225A), 60.5% (N262A), and 100% (Y330A), in addition to the complete inactivation of the catalytic residues by the mutation.

A twisted-boat intermediate conformation of the sugar at the − 1 subsite is required for the catalysis of GH5 enzymes [35]. In the *Tf*Cel5A-substrate complex, the sidechains of the two absolutely conserved residues 225H and 262N formed multiple hydrogen bonds with the O3ʹ-hydroxyl group of the sugar at the − 1 subsite (Fig. 4c). 225H and 262N were located at loops 3 and 4, respectively. Interestingly, the MD simulations revealed that loop 3 became more flexible following substrate binding, whereas loop 4 remained rigid in the absence or presence of the substrate (Fig. 2b). Because of the difference in the flexibility of loops 3 and 4, 225H moved frequently, whereas 262N remained immobile. Therefore, the stable hydrogen bonding interaction formed between 262N and the sugar at the − 1 subsite might function as an “anchor” of the sugar, whereas the severe fluctuation of 225H led to the distortion of sugar conformation at the − 1 subsite. The experimental results of H225A and N262A mutants and the conservation of both residues in the GH5 family further confirmed their key roles in the catalysis of GH5 cellulases.

### Stabilization of the catalytic residues

Following the formation of the intermediate, two catalytic residues (e.g., 263E and 355E in *Tf*Cel5A) initiated the hydrolysis of the glycosidic bond between the sugars at the − 1 and + 1 subsites, which required the precise coordination of the catalytic residues. We found that 328H and 330Y on β-sheet 6 of *Tf*Cel5A were close to the catalytic residues and highly conserved in the GH5 family (Fig. 1c). In the MD simulations, 328H and 330Y formed two hydrogen bonds with the O2ʹ-hydroxyl group of the sugar at the − 1 subsite and formed two hydrogen bonds with the sidechain of the nucleophile residue 355E (Fig. 4d). Importantly, 328H and 330Y showed low flexibility during the simulations, which stabilized the carboxyl group of 355E and ensured the precise orientation of the catalytic residues. This explained the significant decrease in the catalytic activity of the Y330A mutant. Additionally, the phenolic hydroxyl group of 330Y helps regulate the deprotonated state of the carboxyl group of 355E with the support of 185R [36]. Taken together, these results indicated that 330Y in loop 6 assisted the precise positioning and protonation state of the nucleophile residues during the catalysis of *Tf*Cel5A.

### Loop 7 regulated product release

**Fig. 5:**
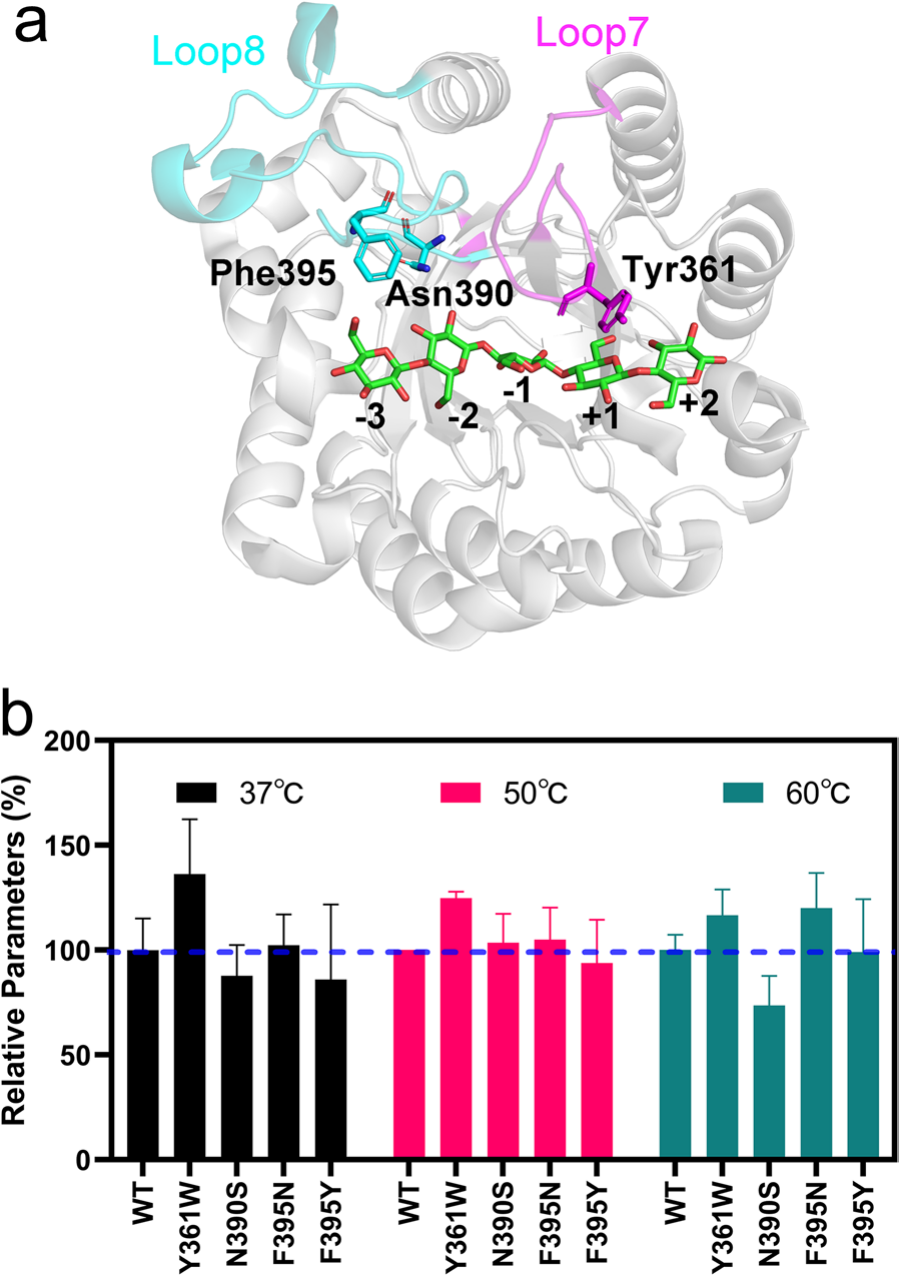
Comparison of catalytic activities between the WT and its mutants at 37 °C, 50 °C, and 60 °C. **a** The amino acid positions selected for site-directed mutations. Loops 7 and 8 are shown in red and cyan, respectively. The subsites of the active site are labeled from − 3 to + 2. **b** The relative catalytic activity of *Tf*Cel5A mutants at different temperatures

After the hydrolysis of the glycosidic bond between the sugars at the − 1 and + 1 subsites, the enzyme needs to release the cleaved oligosaccharide product from the active site and initiate the next catalytic reaction. Interestingly, we found that loop 7 of GH5_2 cellulases was often highly flexible in the substrate-free state but remained stable in the substrate-bound state (Fig. 2b). Notably, Loop 7 interacts with the substrate via a CH-π interaction between 361Y and the pyranose. 361Y is not conserved in the GH5 family and the alanine substitution of 361Y exerted a minor effect on the catalytic activity (Figs. 1c and 4b). We then performed a series of mutations targeting the non-conserved amino acids at loops 7 and 8 (Fig. 5a). Unlike the amino acid mutants at loop 8, the activity of Y361W increased by approximately 40% as compared to that of the WT at 37 °C and was approximately 20% higher than that of the WT at 50 °C. At 60 °C, the mutations of Y361W and F395N increased the catalytic activity by approximately 16% and 20%, respectively (Fig. 5b). Considering that product release is a key limitation of enzyme catalysis at low temperatures [37], the measurements suggest that the mutation of Y361W benefited the release of the product and thereby significantly enhanced the activity at low temperatures. Interaction energy calculation showed the order of binding force was WT > Y361W > Y361A (Additional file 1: Fig. S6). These results indicated that depending on the swing motion of loop 7, the conformational transition might promote the release of the hydrolyzed product from the reducing end of the active site of *Tf*Cel5A.

## Discussion

GH5 cellulases are the key components of enzyme cocktails used for hydrolyzing lignocellulosic biomass in modern biorefinery industries. Generally, a classical two-step, retaining mechanism is used to achieve the catalysis [35, 38]. Upon substrate binding to the active site of the enzymes, a glycosyl-enzyme intermediate is rapidly formed through the glycosylation process. Subsequently, the nucleophile residue (e.g., E355 in *Tf*Cel5A) and the acid/base residue (e.g., E263 in *Tf*Cel5A) use a water molecule to attack the anomeric carbon of the sugar at the − 1 subsite and finally cleave the glycosidic bond [15, 39]. Despite the basic understanding of the catalytic mechanism of cellulases [40], the dynamic behavior of key loops in active site architecture and their functions in substrate binding, catalysis and product release are relatively little studied.

**Fig. 6:**
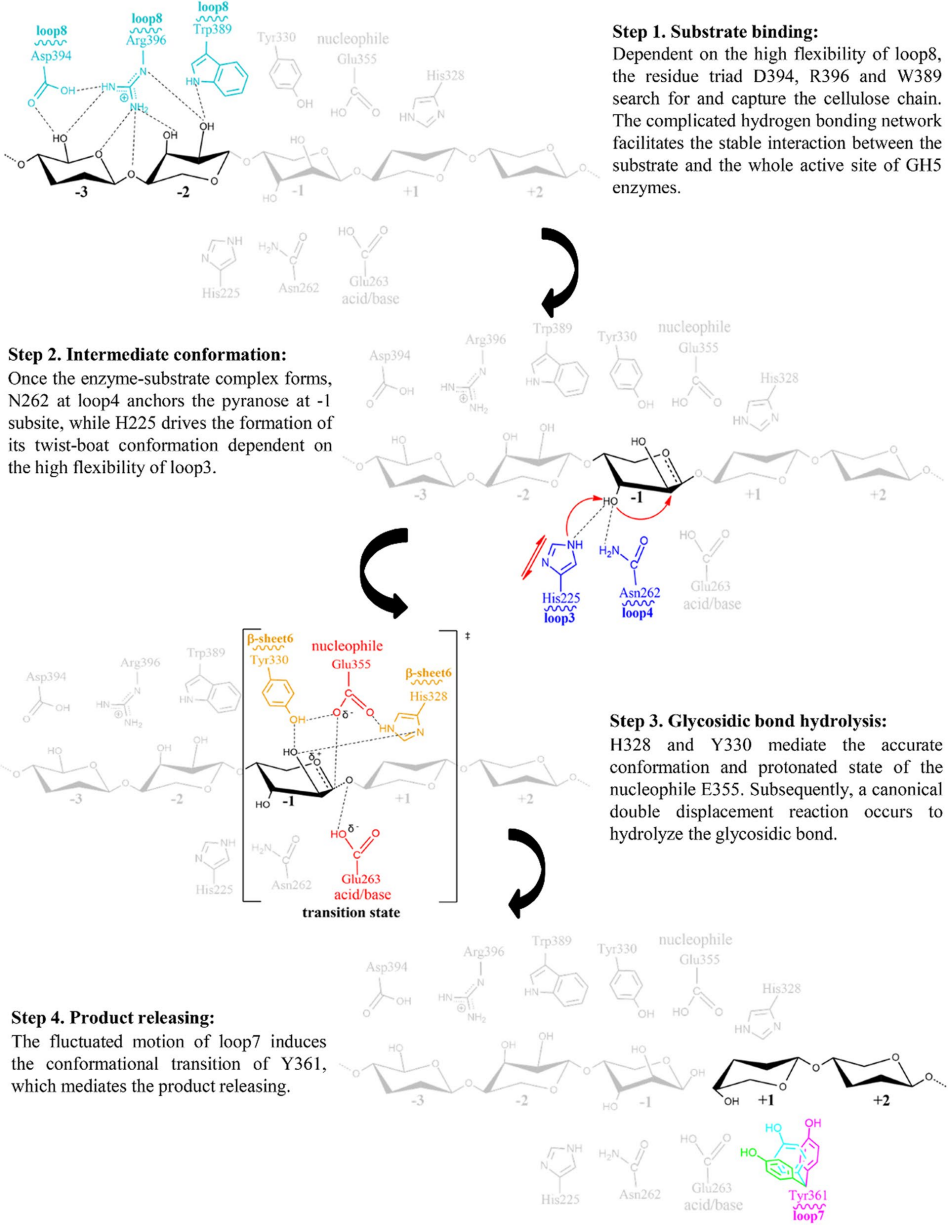
A conceptual model to elucidate the catalysis of GH5_2 cellulases

An in-depth analysis of the active site architecture is a useful strategy to clarify the catalytic mechanism [41–43]. The unique spatial arrangement of the amino acid residues in the enzyme active site determines the specificity and efficiency of substrate degradation [44]. Using GH5_2 cellulases as a model, we systematically investigated their structure-catalysis relationship by integrating multiscale approaches and proposed a theoretical model of the entire catalytic process (Fig. 6).

At the − 3 and − 2 subsites, depending on the high flexibility of loop 8, a residue triad (389W, 394D, and 396R in *Tf*Cel5A) functioned as a “grapple-hook” to search for and capture the cellulose chain into the active cleft. Given that the residue triad sits at the entrance of the active site, the interaction of the triad with the substrate might trigger the subsequent binding of the substrate to the enzyme cleft in a zipper-like manner (Fig. 6, Step 1). The experiments of the site-directed mutations at 390N and 395F demonstrated the availability of potential chemical space to optimize the sequence motif of loop 8 for better catalytic activity (Fig. 5b). This provides a promising direction to improve the enzyme activity by modifying the architecture at the − 3 and − 2 subsites of the active site [37, 45]. When the enzyme–substrate complex was formed, the flexibility of loop 3 substantially increased, and 225H contributed to the hydrogen bonding interaction with the O3′-hydroxyl group of the sugar at the -1 subsite (Fig. 4c). Through the anchoring effect of 262N at loop 4, the vibration of 225H strongly stimulated the transition of the sugar ring from a chair conformation to a twist-boat conformation (Fig. 6, Step 2), which mediated the formation of a glycosyl-enzyme intermediate [35]. Subsequently, the glycosidic bond between the sugars at the − 1 and + 1 subsites was cleaved, which requires the precise orientation and protonation state of the catalytic residues [15]. Our results revealed that 328H and 330Y stabilized the side chain of the nucleophile residue (Fig. 6, Step 3) and finally achieved the hydrolysis reaction in cooperation with the acid/base residue [40]. Although the residues at the + 1 and + 2 subsites were non-conserved in the GH5 family, the motion of loop 7 facilitated product release by generating steric repulsion between the enzyme and the hydrolyzed product (Fig. 6, Step 4). The sequence diversity of loop 7 might reflect the requirement of GH5_2 cellulases to release the product under different conditions (e.g. under different temperatures and pH) [46]. The significantly increased activity of the mutant Y361W (Fig. 5b) demonstrated that loop 7 was an intriguing target to accelerate product release and thus improve the catalytic activity of GH5_2 cellulases.

## Conclusion

To the best of our knowledge, the present study is the first systematic structure-catalysis relationship study for GH5_2 cellulases. By integrating experimental and computational approaches, we identified the key determinants of the catalytic activity of GH5_2 cellulases. Specifically, loops 3, 7, and 8 at the different subsites of the active site showed unique structural dynamic features, which conferred distinct functions, including substrate binding, intermediate formation, stabilization of catalytic residues, and product release. Most functional residues identified in *Tf*Cel5A were highly conserved in the GH5_2 family; moreover, our design of *Tf*Cel5A mutants suggested that the remote subsites provide intriguing chemical space for future engineering of GH5_2 cellulases. Collectively, our results refreshed the understanding of the catalytic mechanism of GH5_2 cellulases and guided the rational design of superior biocatalysts for biomass conversion.

## Material and methods

### Materials

*Escherichia coli* T1 (TransGen, Beijing, China) was used for gene cloning and sequencing. *E. coli* BL21 (DE3) (Invitrogen, Carlsbad, CA, USA) was used as a heterologous expression host. The *pEASY*-blunt E1 vector (TransGen) was used for constitutive expression in *E. coli*. The *tfcel5A* gene was chemically synthesized (Genewiz, Suzhou, China). The recombinant plasmid *pEASY*-blunt E1/*tfcel5A*, harboring the endoglucanase-encoding gene *tfcel5A* and a 6 × His-tag at the C-terminal, was constructed to express the corresponding *Tf*Cel5A protein (GenBank accession no. Q01786.2). All chemicals were of analytical grade.

### Mutagenesis of *Tf*Cel5A

A Fast Mutagenesis System Kit purchased from TransGen was used to perform site-directed mutagenesis in accordance with the manufacturer’s instructions. The recombinant plasmid *pEASY*-blunt E1/*tfcel5A* was used as the PCR template. Mutagenic primers were synthesized by Sangon (Shanghai, China), and their sequences are summarized in Additional file 1: Table S1.

### Enzyme expression and purification

*Escherichia coli* BL21 (DE3) cells were cultured in LB medium supplemented with 50 μg/mL kanamycin at 37 °C until the optical density of the medium reached 0.6–0.8 at 600 nm. Subsequently, for protein induction, isopropyl-β-D-thiogalactopyranoside (IPTG; Solarbio, Beijing, China) was added to the medium at the final concentration of 50 mM. The culture was incubated for 20 h in a shaker (20 °C, 200 rpm). After centrifugation, the precipitate was harvested and resuspended in a lysis buffer (50 mM NaH_2_PO_4_, 300 mM NaCl, pH 8.0). Ni^2+^ affinity chromatography (HisTrap™ FF crude; GE Healthcare, Buckinghamshire, UK) was used for protein purification after ultrasonic fragmentation. The eluent was replaced with PC buffer (20 mM sodium phosphate, 10 mM citrate, pH 6.0) and subjected to ultrafiltration through a 3-kDa molecular cutoff membrane (Millipore, Billerica, MA, USA) at 4 °C. SDS-PAGE was performed on a 12% (*w*/*v*) polyacrylamide gel, and the protein bands were stained with Coomassie Brilliant Blue R-250 (Sigma–Aldrich, St. Louis, MO, USA). Protein concentration was determined using the classical Bradford method [47].

### Enzymatic property assay

CMC-Na (400–800 centipoises in water at room temperature) was purchased from Sigma–Aldrich as a substrate for enzymatic property assay. The reaction system comprised 150 µL of 1% (*w*/*v*) CMC-Na and 15 µg of the purified enzyme in a 300 µL reaction mixture. The reaction mixture was incubated at 60 °C and pH 5.0 for 30 min, and the reaction was terminated by the addition of 300 µL of 3,5-dinitrosalicylic acid (DNS) reagent in a boiling water bath for 10 min. After the sample was cooled-down to ambient temperature, the absorbance was measured at 540 nm [48]. One unit of enzyme activity (U) was defined as the amount of enzyme (mg) that released 1 μmol of glucose per minute under the optimal assay conditions. The enzyme activities of WT and variants are expressed as mean ± SD (n = 3). The relative parameters of WT were set to 100%. The bars indicate the standard errors.

Fluorescent spectrometry [49]was used to detect the substrate binding constant (Ka). Briefly, 100 µL of the purified enzyme and 100 µL of acetate buffer (50 mM, pH 5.0) were added to a colorimeter tube. The excitation wavelength was 295 nm, and the emission wavelength ranged from 300 to 500 nm. Kinetic parameters were measured using an appropriate equivalent amount of the enzyme diluted with varying concentrations of CMC-Na (1–10 mg/mL). The reaction was performed in 50 mM acetate buffer (pH 5.0) at 60 °C for 30 min. Each experiment was performed in triplicate. The relative parameters of WT were set to 100%. The bars indicate the standard errors.

### Analysis of loops in the active site architecture of GH5_2 subfamily

All protein crystal structures used in the present study were obtained from the Protein Data Bank (PDB) archive (http://www.rcsb.org). Homologous sequence alignments were performed using CLUSTAL [50]. Phylogenetic trees were constructed by the neighbor-joining method using MEGA7.0 [51] and optimized by iTOL online software [52]. Sequence profiling of the active site architecture of the GH5_2 subfamily was performed by the online software WebLogo [53]. All structural diagrams were drawn using PyMOL software (https://pymol.org/). The b-factor of the enzyme molecule is recorded in the atomic coordinate file of the PDB structure and can be displayed by PyMOL. Temperature coloring is used to visualize the uncertainty of each atom.

### MD simulations

GROMACS 4.5 was used to perform all-atom simulations for *Tf*Cel5A and additional 21 GH5 cellulases. Two MD-simulation systems: substrate-free state and substrate-bound state were constructed to investigate the dynamic behavior of the enzyme *Tf*Cel5A. *Tf*Cel5A binds cellopentose in a substrate-bound system. The cellopentose was removed from the complex to keep the dissociated state in substrate-free system. The protein was solvated using SPC water models in a cubic box, and 0.1 M sodium chloride was used to neutralize the system. Van der Waals and electrostatic interaction energies between the enzyme and the substrate were calculated using the Amber force field equation [54]. The simulation details were refined to match unbiased MD simulations [55, 56].

### Supplementary Information

**Additional file 1: Table S1.** Primers of *Tf*Cel5A mutants. **Figure S1.** Phylogenetic tree of GH5 main subfamilies. **Figure S2.** Changes in the structural flexibility of GH5_2 enzymes before and after substrate binding. Root Mean Square Fluctuation (RMSF) values were calculated by molecular dynamics simulations. PDB codes of the used enzymes are labeled. **Figure S3.** Structural flexibility of enzymes from major GH5 subfamilies. The PDB codes of the enzymes are labeled. Visualizing the root mean square fluctuation changes in atomic positions is done by coloring by RMSF value. Atoms with low RMSF values are colored blue, while atoms with high RMSF values are colored red. **Figure S4.** The hydrogen bonding network constructed by the triplet residues at − 3 and − 2 subsites. **a** The wild-type *Tf*Cel5A (PDB: 2CKR). **b** *Bacillus subtilis* endo-1,4-beta-glucanase (PDB: 3PZT). **c** *Salipaludibacillus agaradhaerens *endoglucanase Cel5A (PDB: 1H11). **d** *Dickeya dadantii *cellulase Cel5 (PDB: 1EGZ). **Figure S5.** Measurements of catalytic activity and substrate binding affinity of the loop 1, 2, and 8 mutants. **Figure S6.** The interaction energy of WT, Y361A and Y361W mutants with ligand, respectively.

## Data Availability

The data generated or analyzed during this study are included in this published article and its additional files. Further datasets used and analyzed during the current study are available from the corresponding author on reasonable request.

## Abbreviations

GH: Glycoside hydrolase
CAZy: Carbohydrate-active enZYmes;
MD: Molecular dynamics
RMSF: Root mean square fluctuation
PDB: Protein Data Bank
IPTG: Isopropyl-β-d-thiogalactopyranoside
DNS: 3,5-dinitrosalicylic acid
