# Supplementary material for: Dynamics of loops surrounding the active site architecture in GH5_2 subfamily TfCel5A for cellulose degradation
Source: Biotechnol Biofuels Bioprod. 2023 Oct 18;16:154. doi: 10.1186/s13068-023-02411-2 (PMC10583438; doi:10.1186/s13068-023-02411-2)
Supplement: Supplementary file 1 — Additional file 1: Table S1. Primers of TfCel5A mutants. Figure S1. Phylogenetic tree of GH5 main subfamilies. Figure S2. Changes in the structural flexibility of GH5_2 enzymes before and after substrate binding. Root Mean Square Fluctuation (RMSF) values were calculated by molecular dynamics simulations. PDB codes of the used enzymes are labeled. Figure S3. Structural flexibility of enzymes from major GH5 subfamilies. The PDB codes of the enzymes are labeled. Visualizing the root mean square fluctuation changes in atomic positions is done by coloring by RMSF value. Atoms with low RMSF values are colored blue, while atoms with high RMSF values are colored red. Figure S4. The hydrogen bonding network constructed by the triplet residues at − 3 and − 2 subsites. a The wild-type TfCel5A (PDB: 2CKR). b Bacillus subtilis endo-1,4-beta-glucanase (PDB: 3PZT). c Salipaludibacillus agaradhaerens endoglucanase Cel5A (PDB: 1H11). d Dickeya dadantii cellulase Cel5 (PDB: 1EGZ). Figure S5. Measurements of catalytic activity and substrate binding affinity of the loop 1, 2, and 8 mutants. Figure S6. The interaction energy of WT, Y361A and Y361W mutants with ligand, respectively. [file 13068_2023_2411_MOESM1_ESM.docx]

**Additional file to**

**Dynamics of loops surrounding the active site architecture in GH5****_2 subfamily *Tf*Cel5A for cellulose degradation**

Xiuyun Wu^1^, Sha Zhao^1^, Zhennan Tian^1^, Chao Han^3^, Xukai Jiang^2^, and Lushan Wang^1^*

^1^State Key Laboratory of Microbial Technology, Institute of Microbial Technology, Shandong University, Qingdao, 266237, China

^2^National Glycoengineering Research Center, Shandong University, Qingdao, 266237, China

^3^Shandong Key Laboratory of Agricultural Microbiology, Shandong Agricultural University, Tai’an, 271018, China

Corresponding author:

Lushan Wang

State Key Laboratory of Microbial Technology, Institute of Microbial Technology, Shandong University, Qingdao

Email: lswang@sdu.edu.cn

**Table S1.** Primers of *Tf*Cel5A mutants.

| **Primer** | **Sequence (from 5’ to 3’)** |
| --- | --- |
| F163A sense | ATCCAGTGGGCAGACCACTGCCTGA |
| F163A antisense | TCAGGCAGTGGTCTGCCCACTGGAT |
| D394A sense | CAAGTGGAACTACTCGGACGCATTC |
| D394A antisense | GAATGCGTCCGAGTAGTTCCACTTG |
| F395A sense | GAACTACTCGGACGACGCACGTTCC |
| F395A antisense | GGAACGTGCGTCGTCCGAGTAGTTC |
| W162A sense | ATCCAGGCATTCGACCACTGCCTGA |
| W162A antisense | TCAGGCAGTGGTCGAATGCCTGGAT |
| Y189A sense | GCCTGTCCATGGCTATCCAGGAAGA |
| Y189A antisense | GTCTTCCTGGATAGCCATGGACAGG |
| E192A sense | CCATGTACATCCAGGCAGACGGCTA |
| E192A antisense | GTAGCCGTCTGCCTGGATGTACATG |
| H158A sense | ATGAGCACCGCAGGCATCCAGTGGT |
| H158A antisense | ACCACTGGATGCCTGCGGTGCTCAT |
| W389A sense | GTGGACCAAGGCAAACTACTCGGAC |
| W389A antisense | GTCCGAGTAGTTTGCCTTGGTCCAC |
| N390A sense | GTGGACCAAGTGGGCATACTCGGAC |
| N390A antisense | GTCCGAGTATGCCCACTTGGTCCAC |
| R396A sense | CTACTCGGACGACTTCGCATCCGGC |
| R396A antisense | GCCGGATGCGAAGTCGTCCGAGTAG |
| H225A sense | GTACGTGATCGTGGACTGGGCAATC |
| H225A antisense | GATTGCCCAGTCCACGATCACGTAC |
| L227A sense | GTGGACTGGCACATCGCTACCCCGG |
| L227A antisense | CCGGGGTAGCGATGTGCCAGTCCAC |
| N262A sense | GTGCTCTACGAGATCGCCGCAGAAC |
| N262A antisense | GTTCTGCGGCGATCTCGTAGAGCAC |
| H328A sense | ACATCATGTACGCCTTCGCATTCTA |
| H328A antisense | TAGAATGCGAAGGCGTACATGATGT |
| Y330A sense | CTTCCACTTCGCTGCGGCCTCGCAC |
| Y330A antisense | GTGCGAGGCCGCAGCGAAGTGGAAG |
| E263A sense | CTACGAGATCGCCAACGCACCCAAC |
| E263A antisense | GTTGGGTGCGTTGGCGATCTCGTAG |
| E355A sense | GGTCTTCGTCACCGCATTCGGCACC |
| E355A antisense | GGTGCCGAATGCGGTGACGAAGACC |
| H334A sense | CTCGGCACGCGACAACTACCTCAAC |
| H334A antisense | GTTGAGGTAGTTGTCGCGTGCCGAG |
| W299A sense | GCGGCGCTTCGTCGCTCGGCGTCTC |
| W299A antisense | GAGACGCCGAGCGACGAAGCGCCGC |
| Y361A sense | GAGTTCGGCACCGAGACCGCTACCG |
| Y361A antisense | CGGTAGCGGTCTCGGTGCCGAACTC |
| S305A sense | GTCGCTCGGCGTCGCAGAAGGCTCC |
| S305A antisense | GGAGCCTTCTGCGACGCCGAGCGAC |
| Y338A sense | ACCGCGACAACGCTCTCAACGCGCT |
| Y338A antisense | AGCGCGTTGAGAGCGTTGTCGCGGT |
| D394K sense | CAAGTGGAACTACTCGGACAAATTC |
| D394K antisense | GAATTTGTCCGAGTAGTTCCACTTG |
| R396E sense | GAACTACTCGGACGACTTCGAATCC |
| R396E antisense | GGATTCGAAGTCGTCCGAGTAGTTC |
| D394AR396A sense | CTACTCGGACGCATTCGCATCCGGC |
| D394AR396A antisense | GCCGGATGCGAATGCGTCCGAGTAG |
| D394KR396E sense | CTACTCGGACAAATTCGAATCCGGC |
| D394KR396E antisense | GCCGGATTCGAATTTGTCCGAGTAG |


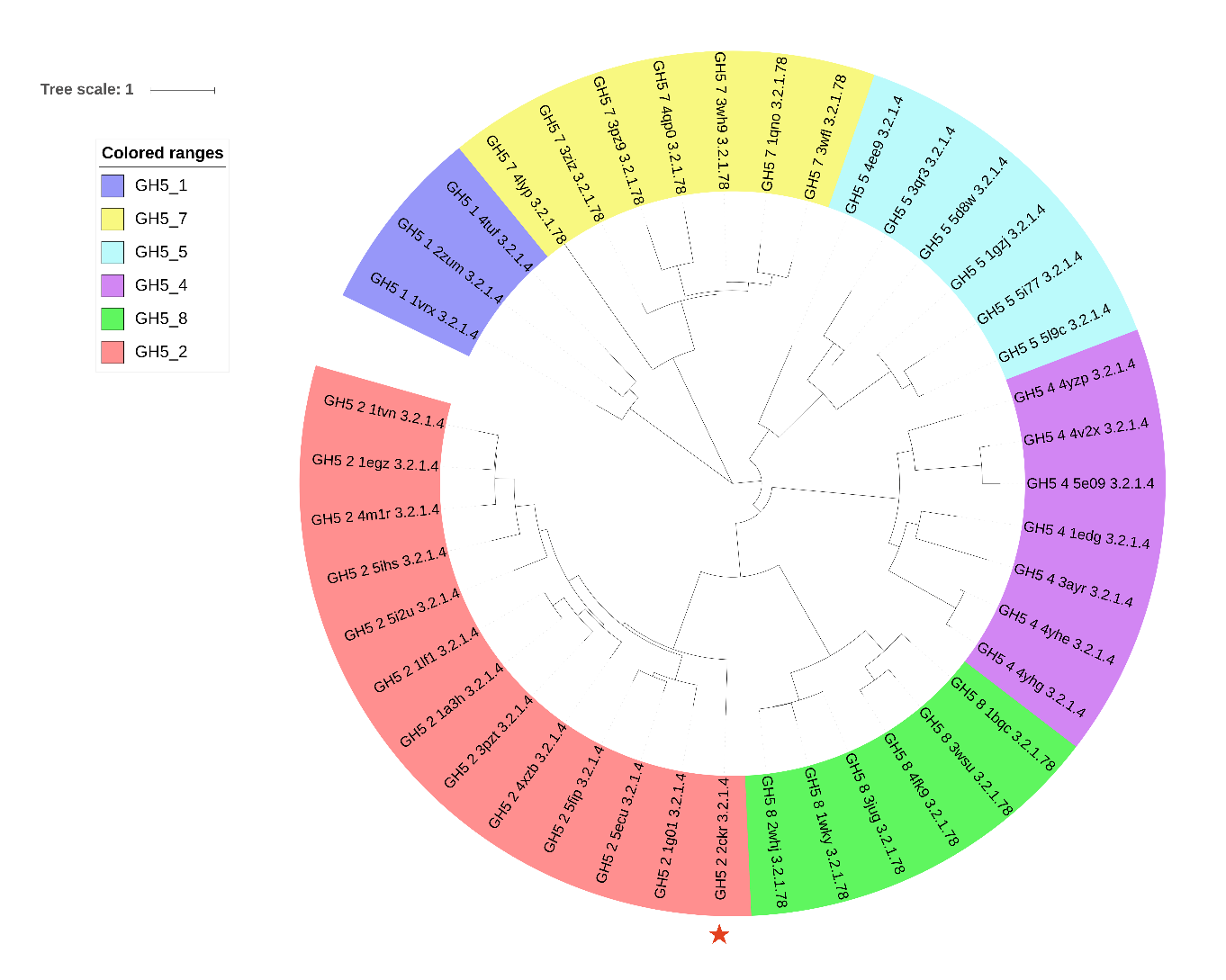


**Figure S1.** Phylogenetic tree of GH5 main subfamilies


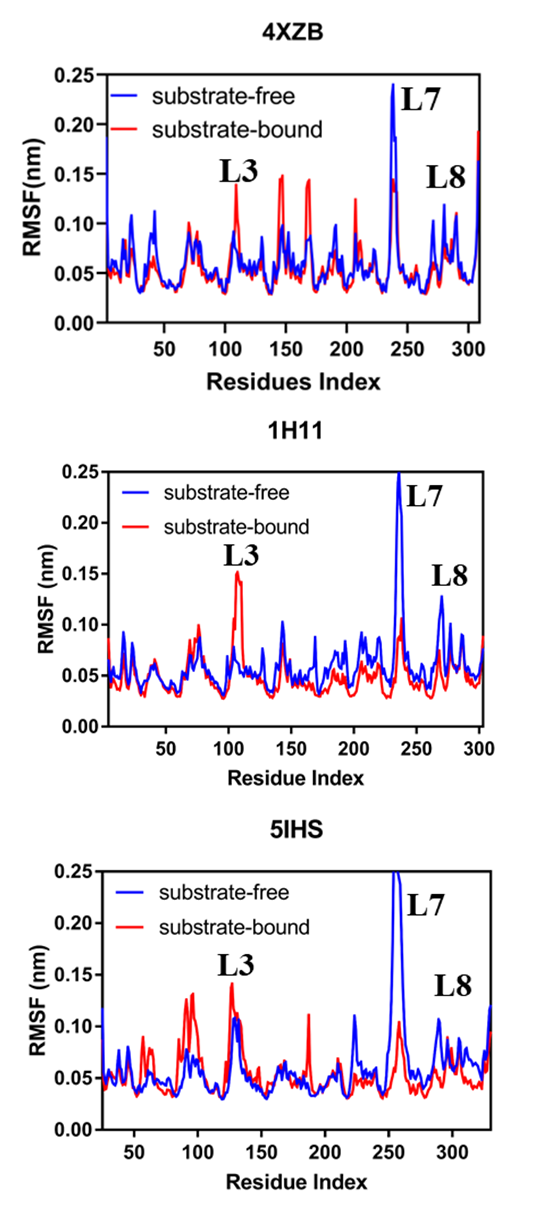


**Figure S2.** Changes in the structural flexibility of GH5_2 enzymes before and after substrate binding. Root Mean Square Fluctuation (RMSF) values were calculated by molecular dynamics simulations. PDB codes of the used enzymes are labeled.


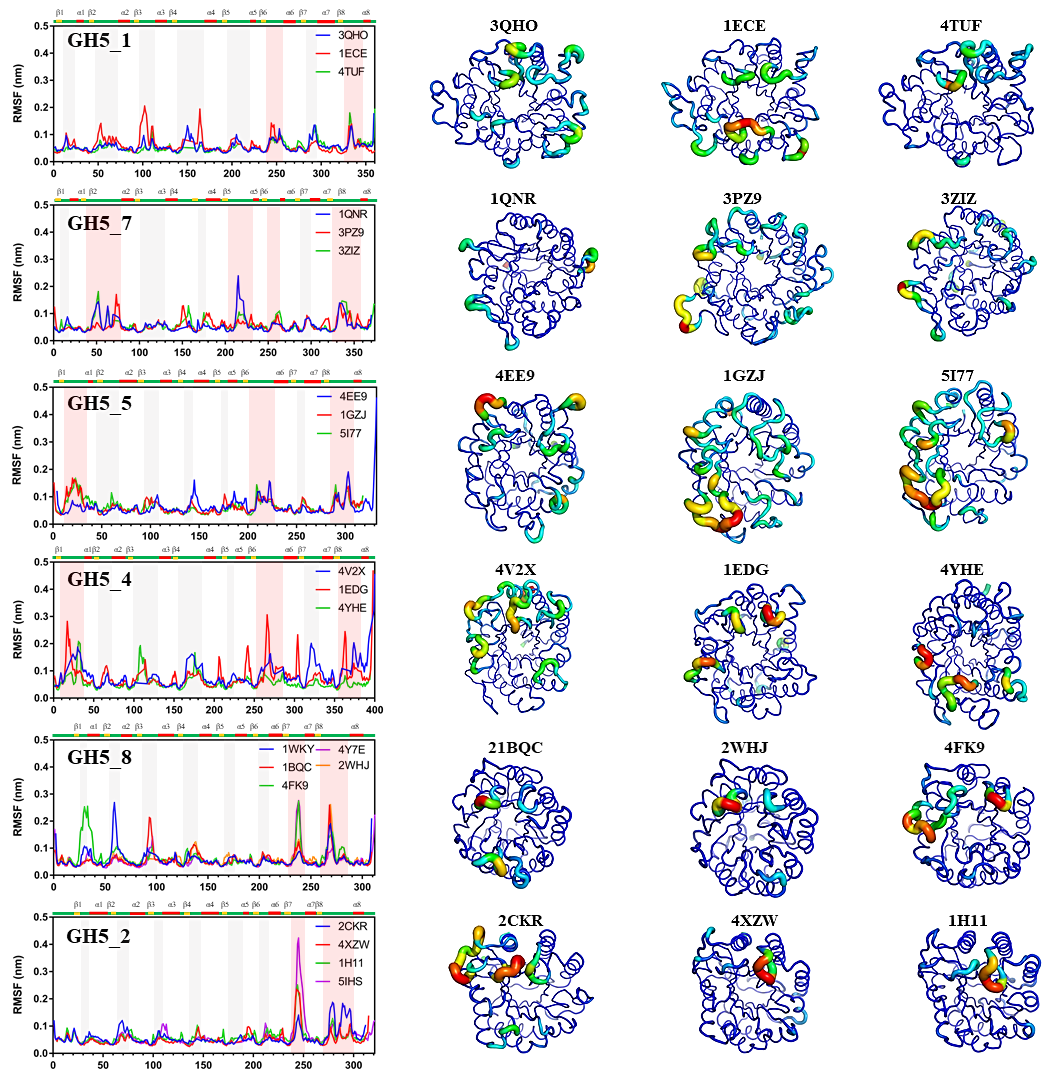


**Figure S3.** Structural flexibility of enzymes from major GH5 subfamilies. The PDB codes of the enzymes are labeled. Visualizing the root mean square fluctuation changes in atomic positions is done by coloring by RMSF value. Atoms with low RMSF values are colored blue, while atoms with high RMSF values are colored red.

**
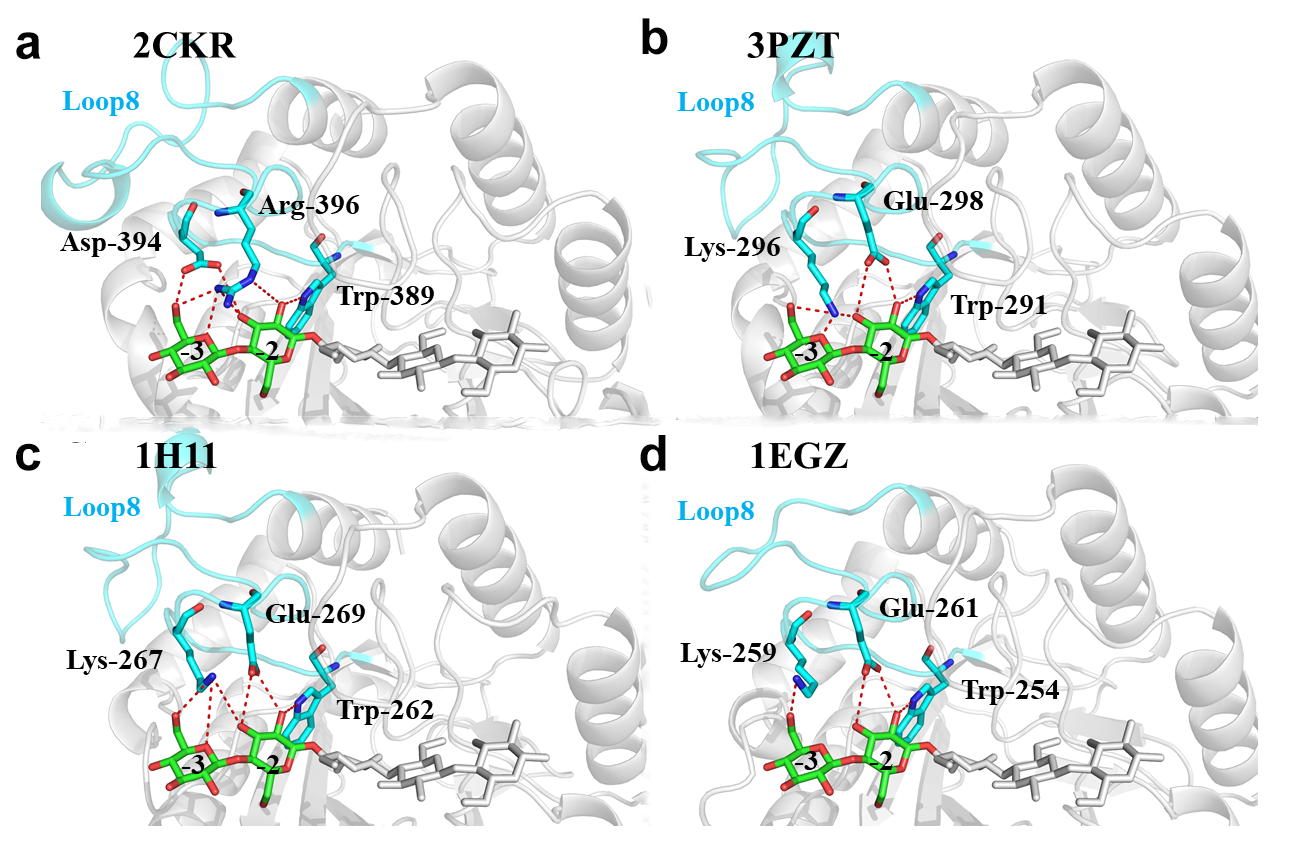
**

**Figure S4.** The hydrogen bonding network constructed by the triplet residues at -3 and -2 subsites. (a) the wild-type *Tf*Cel5A (PDB: 3CKR). (b) *Bacillus subtilis* endo-1,4-beta-glucanase (PDB: 3PZT). (c) *Salipaludibacillus agaradhaerens* endoglucanase Cel5A (PDB: 1H11). (d) *Dickeya dadantii* cellulase Cel5 (PDB: 1EGZ).


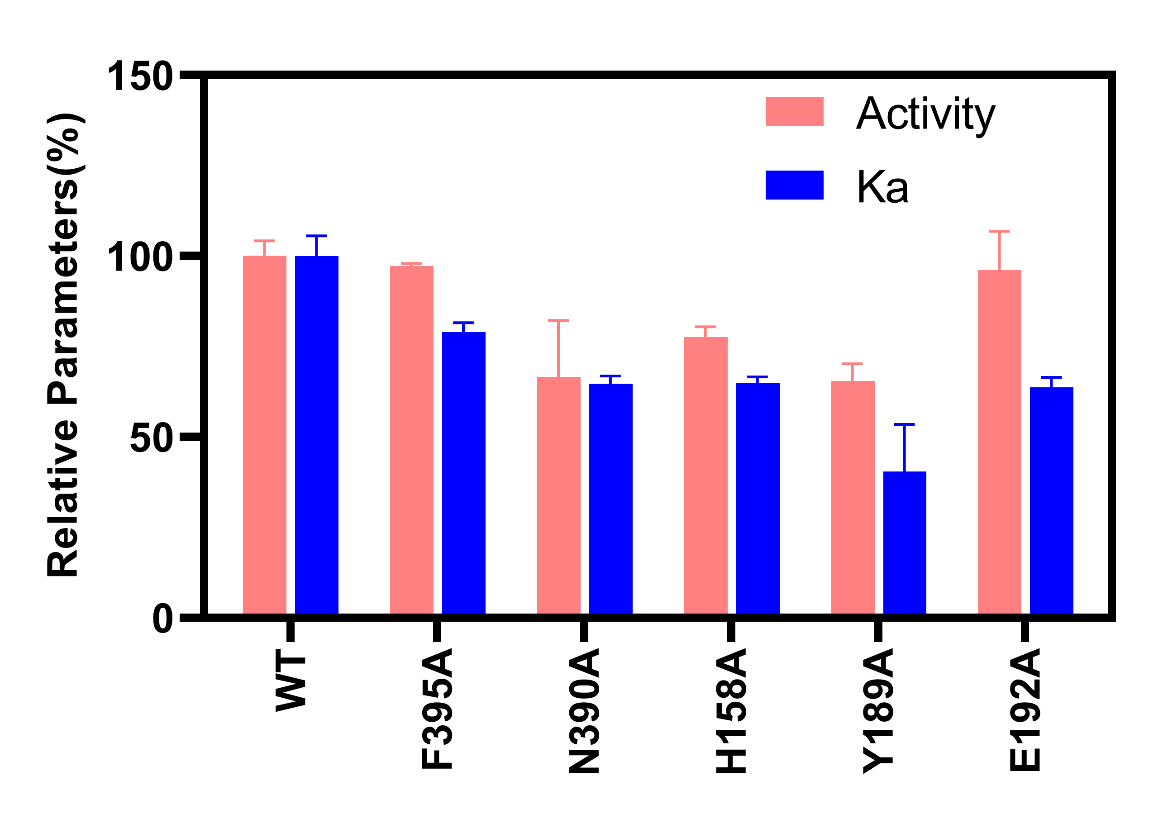


**Figure S5.** Measurements of catalytic activity and substrate binding affinity of the loop 1, 2, and 8 mutants.


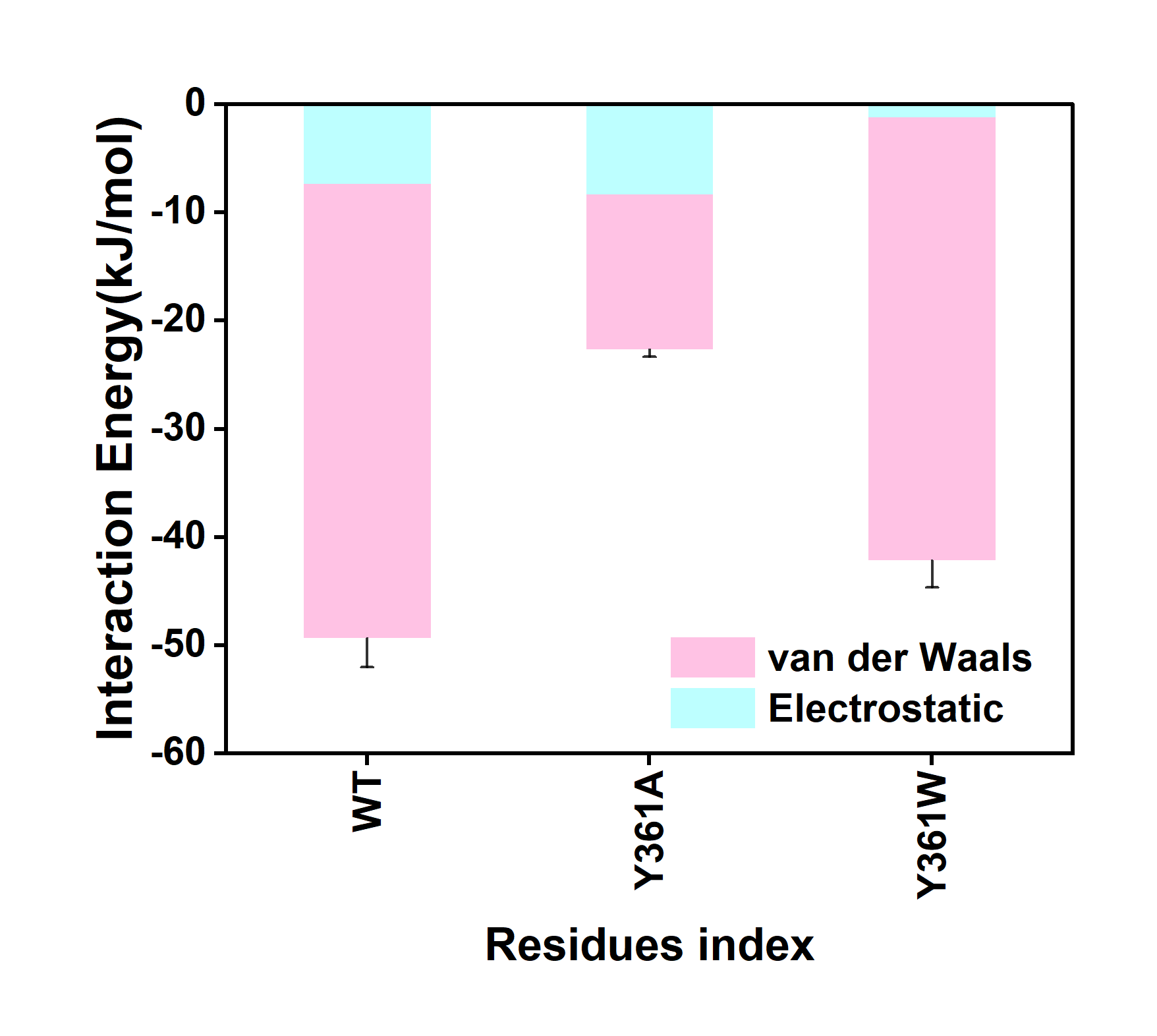


**Figure S6.** The interaction energy of WT, Y361A and Y361W mutants with ligand, respectively.
